# P282: Health care exposure prevention of tuberculosis by successful implementation of employee safety policy- our experience

**DOI:** 10.1186/2047-2994-2-S1-P282

**Published:** 2013-06-20

**Authors:** M Sri Ratnamani, R Rao

**Affiliations:** 1 Microbiology, apollo hospitals, Hyderabad, India

## Introduction

India accounts for one-fifth of the global Tuberculosis (TB) incident cases. It is estimated that annually around 330,000 Indians die due to TB.[1] The risk of exposure to Health care workers (HCW) is often overlooked and the hospitals also underplay it. In one of the data published in Lung India 11.2 new cases per 1000 person-years of exposure was reported.[2,3] It is an important challenge everywhere, particularly in low and low to middle income countries. [4]

## Objectives

A TB infection control plan that outlines a protocol for the prompt recognition, separation and investigation for suspected or confirmed TB disease was formulated as part of employee safety policy.

## Methods

Our TB infection control program was based on a three-level hierarchy of control measures based on CDC recommendations 2012. Triage of Suspected TB patients on hospital admission was strictly implemented. We used smear-negative algorithms. **Structural modifications-** Emergency department was provided with a negative pressure room to keep these patients. **Behavioral change -** Till the patient is proved negative, the staff attending the patient wear N95 mask to reduce the risk for exposure.

## Results

Before implementation open TB cases admitted annually without screening were 15, 14, and 15 in 2007, 2008 and 2009 respectively and after Implementation 5,0,2 in 2010,2011 and 2012 respectively.

## Conclusion

Implementation of policies to prevent exposure of health care workers to Tuberculosis is imperative to prevent Nosocomial TB Infection. Through a effective employee safety policy and a active Infection Control team, nosocomial TB spread can be prevented.

## Competing interests

None declared
